# Relevance of internal time and circadian robustness for cancer patients

**DOI:** 10.1186/s12885-016-2319-9

**Published:** 2016-04-21

**Authors:** Elisabet Ortiz-Tudela, Pasquale F. Innominato, Maria Angeles Rol, Francis Lévi, Juan Antonio Madrid

**Affiliations:** Chronobiology Laboratory, Department of Physiology, University of Murcia, IMIB-Arrixaca, Murcia, Spain; INSERM, UMRS 776 « Biological Rhythms and Cancers », Villejuif, France; APHP, Chronotherapy Unit, Department of Oncology, Paul Brousse Hospital, Villejuif, France; Warwick Medical School, Cancer Chronotherapy Unit, Coventry, UK; Chronobiology Laboratory, Department of Physiology, Faculty of Biology, University of Murcia, Campus de Espinardo, Espinardo, Murcia, Zip Code 30100 Spain

**Keywords:** Circadian, gastro-intestinal cancer, personalization, TAP, chemotherapy

## Abstract

**Background:**

Adequate circadian timing of cancer treatment schedules (chronotherapy) can enhance tolerance and efficacy several-fold in experimental and clinical situations. However, the optimal timing varies according to sex, genetic background and lifestyle. Here, we compute the individual phase of the Circadian Timing System to decipher the internal timing of each patient and find the optimal treatment timing.

**Methods:**

Twenty-four patients (11 male; 13 female), aged 36 to 77 years, with advanced or metastatic gastro-intestinal cancer were recruited. Inner wrist surface Temperature, arm Activity and Position (TAP) were recorded every 10 min for 12 days, divided into three 4-day spans before, during and after a course of a set chronotherapy schedule. Pertinent indexes, I < O and a new biomarker, DI (degree of temporal internal order maintenance), were computed for each patient and period.

**Results:**

Three circadian rhythms and the TAP rhythm grew less stable and more fragmented in response to treatment. Furthermore, large inter- and intra-individual changes were found for T, A, P and TAP patterns, with phase differences of up to 12 hours among patients. A moderate perturbation of temporal internal order was observed, but the administration of fixed chronomodulated chemotherapy partially resynchronized temperature and activity rhythms by the end of the study.

**Conclusions:**

The integrated variable TAP, together with the asynchrony among rhythms revealed by the new biomarker DI, would help in the personalization of cancer chronotherapy, taking into account individual circadian phase markers.

## Background

The circadian system controls the timing of all processes in the organism and is composed of a set of structures whose core piece lies in the suprachiasmatic nuclei of the hypothalamus (SCN) [1]. Impairment of the biological clock has been associated with an increased risk of developing several diseases, such as cancer, cognitive impairments, metabolic syndrome, cardiovascular dysfunction and immune dysregulation [2]. Moreover, circadian disruption has been reported to decrease survival of cancer patients [3–5]. This concern is so great that the International Agency for Research on Cancer has classified long-term shift work involving circadian disruption in Group 2A, meaning that it is “probably carcinogenic to humans” [6].

Knowledge of the rhythmic features of drug metabolism, cellular detoxification and cell division cycle [7] has been used to optimize cancer treatments by means of chronotherapy. Chronotherapy mainly involves chronomodulated delivery schedules, and consists of the administration of each drug according to a delivery pattern with precise circadian times in order to achieve the best tolerance and efficacy [8]. However, simpler approaches delivering a conventional treatment either during the morning or during the afternoon have also been reported [9, 10].

Scheduled cancer chronotherapy has proven to be a reliable and advantageous alternative to fixed chemotherapy schedules, at least in men [8, 11]. However, according to previous papers from our group, chemotherapy administration produces an overall disturbance in the rest-activity rhythm, while different patterns of evolution are evident among patients [12]. Furthermore, an effect of sex on the efficacy of the treatment has also been reported [13, 14], suggesting that efficacy could be theoretically improved by personalizing treatment schedules through the use of individual circadian phase markers, as opposed to general schedules common to all patients [7, 8, 11, 12].

However, the introduction of personalized treatments should take into account circadian characteristics, which can differ not only according to the individual person, but also depending on his/her sex, age and chronotype [15–19]. The knowledge of the individual circadian system robustness could in theory, first, allow us to determine which patients might benefit from a chronomodulated therapy and second, to customize the particular timing of the chronotherapy to match up with the personal circadian time.

Nevertheless, assessing the individual circadian status and the severity of chronodisruption is still an unresolved issue, since there are not enough studies on humans attempting to decipher internal timing. Several studies have monitored the rest-activity rhythm as a biomarker of circadian functions in humans (for a review, see [20]), since this can be non-invasively recorded, even for extended periods of time using a wrist-worn accelerometer (actigraph), thus making it suitable for application in the oncology setting [20]. However, it presents certain limitations because of the existence of strong exogenous influences from the type and timing of activity schedules and artifacts, such as those related to bed partner movements or sleeping when travelling in a car or train, etc. [21, 22].

Core body temperature (CBT) is another circadian marker rhythm whose pattern is generated by the suprachiasmatic nuclei, which also acts as an effector involved in the internal coordination of peripheral clocks. However, its evaluation is not problem-free: the CBT rhythm can be determined using a rectal probe connected to an external recorder [23, 24] (a system that is neither safe nor convenient for ambulatory assessment of rhythms in cancer patients) or telemetric pills (which are very expensive and provide records that are too short). Thus, the topic of non-invasive ambulatory monitoring is attracting increasing interest, with distal skin temperature presently constituting a feasible alternative to CBT [25–27]. The peripheral skin temperature pattern is roughly opposite that of CBT: the highest temperatures occur during early night, while the lowest values are seen in the early morning, after awakening [27]; however, it is important to note that this can be masked by position or activity [28].

For this reason, the combination of different complementary circadian markers in the integrated variable TAP (Temperature, Activity, body Position) is less affected by masking factors than individual variables, and minimizes individual recording artifacts. Thus, its use has been reported to estimate the functional status of the circadian system under real life conditions [26] and to assess the sleep-wake cycle, obtaining better estimates than actigraphy alone [29]. Furthermore, TAP has been correlated to the melatonin rhythm, as a M5 calculated on TAP and the Dim Light Melatonin Onset (DLMO), considered to be the most robust phase marker, presented a correlation coefficient of 0.720 (*p* = 0.006) [30]. Moreover, the simultaneous recording of multiple overt rhythms allows us to assess their internal synchrony, constituting a first approach to evaluating whether temporal order is maintained.

The aim of the current study was to evaluate concomitantly multiparametric TAP in cancer patients undergoing fixed-scheduled chronotherapy. The primary endpoint was to evaluate the individual circadian phase and robustness at baseline. Secondary outcomes included the dynamic and continuous evaluation of chemotherapy-induced changes, the degree of internal rhythmic synchronization and the associations between baseline and on-treatment circadian parameters and clinical outcomes. This novel and unique tool provides relevant information needed to precisely optimize chronotherapy based on the patient's individual phase, in order to improve its therapeutic index.

## Methods

### Study design

A descriptive, exploratory and repeated measures design was implemented in order to evaluate the dynamics of three biomarkers (Temperature, Activity and Position) of the Circadian Timing System (CTS) in cancer patients receiving a multidrug, 5-Fluorouracil-based cancer chronotherapy protocol [31]. The three circadian biomarkers were jointly recorded for 12 days, i.e. during a baseline 4-day span before chronotherapy onset, and over the next 8 days, which corresponded to treatment administration and early recovery. This recording was scheduled to take place at least 2 weeks after the previous chemotherapy cycle. The present study was approved by the hospital's internal review board and ethics committee and abided by the Helsinki Declaration of 1975 (revised in 1983). All the subjects were recruited at the Chronotherapy Unit of the Oncology Department in Paul Brousse Hospital (Villejuif, France). After full explanation of the procedure and objectives and before being enrolled in the study, all patients gave their oral consent to participate in it.

### Eligibility criteria

Eligibility criteria included a histological proof of solid cancer at an advanced or metastatic stage. Patients had to be at least 18 years old, with a physician-rated Performance Status (PS) of less than 3 according to the World Health Organization (W.H.O.) classification, and an estimated life expectancy of at least 6 months. Study entry required Grade < 3 in acute clinical and hematological adverse events (National Cancer Institute Common Toxicity Criteria v3.0) and a treatment-free interval of at least 2 weeks. Non-inclusion criteria were persistent grade 3-4 chronic toxicities (except alopecia), symptomatic brain metastases, concomitant severe infection, surgery during the previous month, severe heart dysfunction or ischemic disease and uncontrolled psychiatric illness.

### Chemotherapy regimens

Non-hospitalized patients received one of four set 4-day multidrug chronotherapy protocols selected according to disease type and status [14, 32, 33]. The use of a multichannel portable and time-programmable infusion pump (Mélodie®, Aguettant, France) enabled fixed-time chronomodulated infusions of 5-fluorouracil-leucovorin (5-FU-LV; delivered from 22:15 to 9:45, with peak rate at 4:00), oxaliplatin (l-OHP, delivered from 10:15 to 21:45, with peak rate at 16:00) and/or irinotecan (CPT-11, delivered from 2:00 to 8:00, with peak rate at 5:00) [14, 32, 33]. As indicated, cetuximab, panitumumab, bevacizumab, or docetaxel were administered over 1-2 h in the outpatient clinic, prior to chronotherapy onset.

### Toxicity assessment

Physical examination and toxicity assessment according to NCI CTC-AE v3.0 criteria were performed both at baseline and prior to the administration of the subsequent treatment course. Blood cell counts were obtained before chronotherapy and weekly thereafter, while blood chemistry was determined at baseline and at 2 or 3 weeks. Treatment-induced grade 1 body weight loss and/or grade 2 fatigue were considered to be relevant toxicities, in view of their prior association with both circadian disruption and outcomes of chronotherapy [3, 12, 34].

### Circadian biomarkers

The wrist temperature rhythm was continuously assessed every 10 min for 12 days using a temperature sensor (Thermochron® iButton DS1921H, Dallas, Maxim Integrated). It was attached within a double-sided cotton sport wrist band, with the sensor surface placed over the radial artery of the non-dominant hand [26–28, 30].

The body position and rest-activity rhythms were determined every 30 seconds over the same 12 days by an accelerometer inserted into a sports band. Its x-axis was set parallel to the humerus bone of the non-dominant arm (Hobo® Pendant G Acceleration Data Logger, Massachusetts, Onset Computer Corporation) [26].

Furthermore, patients were asked to complete a diary with their daily activities such as the awakening moment, main meal times, naps and going-to-bed times during the whole study.

### Biomarker data transformation

The integrated TAP variable was computed as described in [26]. Briefly, wrist temperature (T), motor activity (A) and body position (P) data were normalized so that all values ranged between 0 and 1, after removing artifacts. High skin temperature values are usually found at night, when activity counts and position level data are lowest [26]. Therefore, wrist temperature values were inverted so that all three biomarkers appear with a similar pattern of higher counts during daytime. The mean of the normalized variables (T, A and P) was calculated for each subject. TAP values near 1 support a high activation level, as indicated by a low wrist temperature, a high level of activity and a vertical position. In contrast, near-null values correspond to a deep rest state, as indicated by a high wrist temperature, a low activity level and a horizontal position.

### Time series analyses

The multiparametric recordings allowed the computation of the following nine parameters for each individual circadian biomarker and the integrated variable TAP at baseline, during chronotherapy, and during the early recovery period [28, 35]:

- Phase markers, including the mean value and timing of five consecutive hours with the lowest values (VL5 and L5, respectively) and the mean value and timing of ten and five consecutive hours with the highest values (VM10 and M10, and VM5 and M5, respectively). The phase markers allowed the computation of the relative amplitude (RA), which is the difference between the VM10 and VL5, divided by VM10 + VL5. To facilitate comparisons among variables, this parameter was multiplied by ten for wrist temperature.
- Interdaily Stability (IS), which provides an estimated measure of rhythm stability, ranging between 0 (Gaussian noise) to 1 (perfect rhythm stability from one day to the next).
- Intradaily Variability (IV), which is an estimated measure of rhythm fragmentation, with values of 0 indicating a perfectly sinusoidal curve, and 2 Gaussian noise, respectively.

IV, IS and RA were incorporated into a single variable to yield the Circadian Function Index (CFI). CFI can be calculated as described in [26]. It oscillates between 0 (absence of circadian rhythmicity) and 1 (a robust circadian rhythm).

**Fig. 1:**
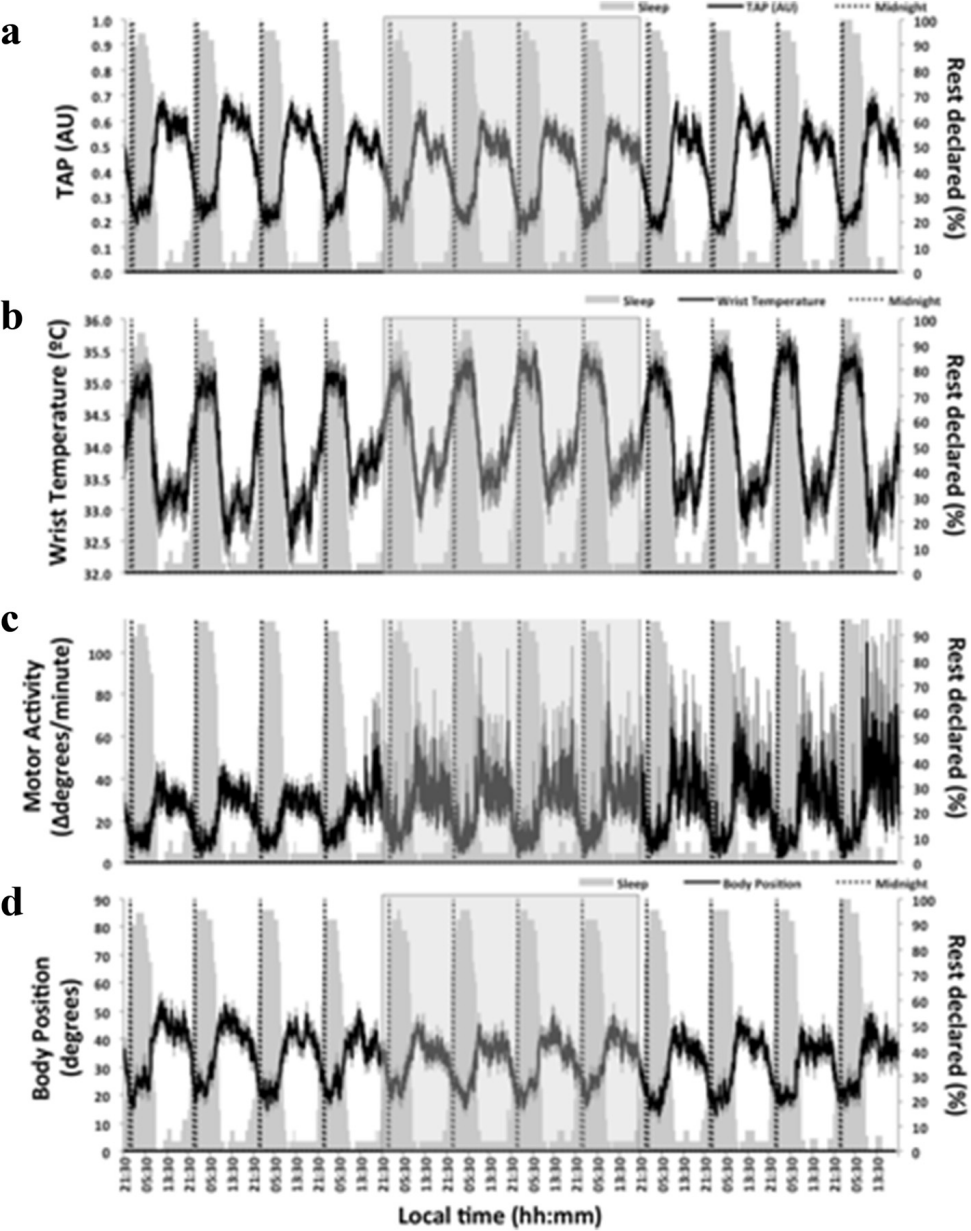
Twelve-day ambulatory mean recording of TAP (**a**), wrist temperature (**b**), motor activity (**c**) and body position (**d**) (*n* = 24) during the study period. The mean of each variable is represented as a black line, and the SEM as a vertical grey line. Rest declared denotes the percentage of patients that declared being asleep at each time point, and is represented as a grey area on each graph for terms of comparison. The chemotherapy period is highlighted by a light grey square

**Fig. 2:**
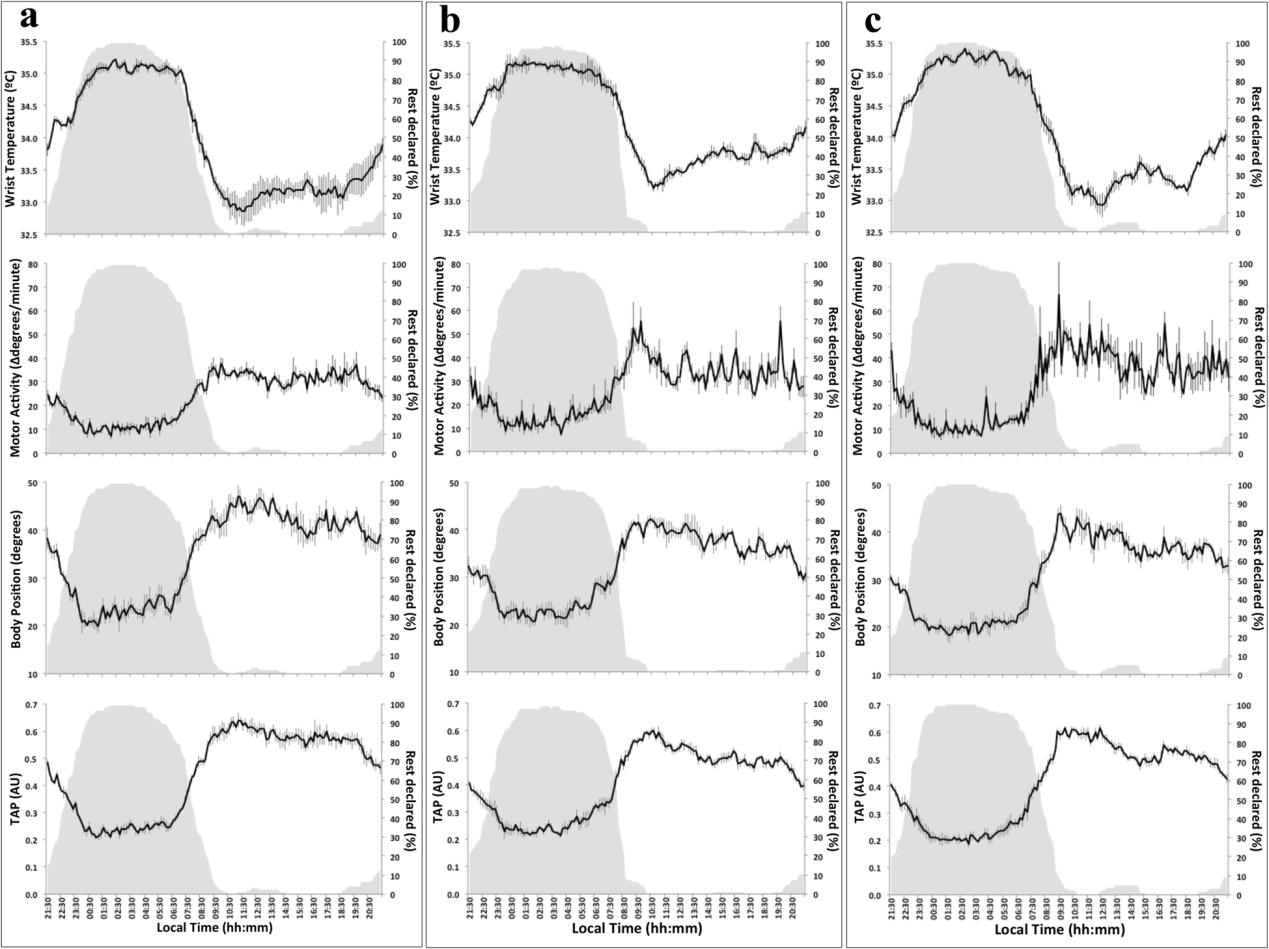
Mean waveforms for each variable and period of time studied (before = **a**, during = **b** and after = **c** receiving chemotherapy). The mean for wrist temperature, motor activity, body position and TAP (*n* = 24) is represented as a black line with SEM as vertical grey lines. Rest declared denotes the percentage of patients that declared being asleep at each time point; this is repeated in grey on each graph for purposes of comparison

Each of these parameters was computed before, after and during chemotherapy. In addition, we generated full study representations (Figs. 1 and 2) of all variables analyzed, as well as mean waveforms for every period of study.

Furthermore, in order to explore the maintenance or alteration of internal temporal order [36] in cancer patients, we calculated the absolute difference between the phase markers M5 (for temperature) and L5 (for activity) for each patient and each period studied. In an ideal situation, both phase markers (midpoint of maximal temperatures and minimal activity) should be synchronized; in the worst case scenario, a 12-hour difference would be found between them. Thus, when differences reached 12 hours, the internal Desynchronization Index (DI) was considered to be 1. When no timing difference was found, DI was 0. Timing differences between 0 and 12 hours were proportionally assigned a value between 0 and 1.

### Statistical analyses

The primary endpoint was the distribution of the circadian phase and robustness parameters at baseline, and their clinical determinants. To this end, descriptive statistics were performed first. Second, differences in T, A, P and TAP parameters at baseline were assessed according to clinical features, using non-parametric tests (Kruskal-Wallis or Mann-Whitney) for ordinal variables (sex, PS, prior chemotherapy) and the Pearson correlation test for quantitative variables (age).

For secondary endpoints, descriptive statistics were obtained for each parameter in every time span. Comparisons between the distribution of the parameters according to time span and sex were made using a repeated measures ANOVA, with sex as a factor (significance level set at *p* < 0.05), followed by Bonferroni *post hoc* pairwise comparisons when appropriate.

Logistic regression was used to explore the predictive value of baseline CFI or DI for the occurrence of clinically relevant toxicities during the same course of chemotherapy. Associations between toxicity and chemotherapy-induced circadian disruption during or after chemotherapy administration were examined using a Kolmogorov-Smirnov *Z* test. Given the exploratory nature of this analysis, the significance level was set at *p* < 0.05.

Wherever appropriate, data in texts and figures are expressed as mean ± SEM. Statistical analyses were performed using the PASW Statistics 18 program (SPSS, IBM, USA).

## Results

### Patient characteristics

Twenty-four patients were enrolled in the study, including 13 women and 11 men aged 36 to 77 years (Table 1). The majority of the patients had metastatic colorectal cancer (21 patients, 88 %), a PS of 0 (18 patients, 75 %) and had received prior chemotherapy (17 patients, 71 %). Sixteen patients (67 %) were given the 4-drug chronotherapy regimen combining irinotecan, oxaliplatin, and 5-FU-LV. Monoclonal antibodies were combined with chronotherapy for 12 patients (50 %).

**Table 1:** Characteristics of patients enrolled in the study

Age	63 y.o. (range: 37 – 78)		
Sex	Male	11	
	Female	13	
Primary Tumor	Colon or rectal	21	
	Pancreas	2	
	Esophagus/cardia	1	
Number of metastatic sites	0	3	
	1	9	
	2	3	
	≥3	9	
WHO-PS	0	18	
	1	6	
	2	0	
Number of prior CT lines	0	7	
	1	2	
	2	6	
	≥3	9	
CT regimen	**Chrono FLO**	1	**3**
	+ CETUXIMAB	1	
	+ DOCETAXEL	1	
	**Chrono IF**	1	**1**
	**Chrono IFL**	/	**4**
	+ BEVACIZUMAB	2	
	+ BEVACIZUMAB + PANITUMUMAB	2	
	**Chrono IFLO**	9	**16**
	+ CETUXIMAB	6	
	+ BEVACIZUMAB	1	

All information was collected before the patients’ inclusion in the study and refers to their previous medical history. Age is expressed as median and range. CT regimen is highlighted in bold. Additional chemotherapy drugs added to the protocol have been marked with (+), whenever necessary

WHO-PS: Performance Status according to the classification of the World Health Organization

CT regimen: protocol followed for chemotherapy treatment. For details, please see the material and methods section

### Baseline circadian biomarkers

Mean waveforms for the baseline period showed coherent and reasonably strong rhythmic patterns for every variable assessed (Fig. 1, Fig. 2 and Table 2). In addition, highest wrist temperature, lowest arm activity, lowest position and highest TAP values occurred in the first half of the night, as indicated by an M5 timing (± SEM) at 03:23 ± 24 min for temperature, an L5 timing at 01:13 ± 42 min for activity, at 01:54 ± 55 min for position and at 02:33 ± 21 min for TAP in the group of 24 patients.

**Table 2:** IS, IV, RA, I < O and CFI calculated for wrist temperature, motor activity, body position and the composite variable TAP rhythms for the whole group (*n* = 24), and for each sex

		Temperature (mean ± SEM)			Motor activity (mean ± SEM)			Body position (mean ± SEM)			TAP (mean ± SEM)		
		Before	During	After	Before	During	After	Before	During	After	Before	During	After
WHOLE GROUP (*n* = 24)	IS	**0.58 ± 0.04** ^**ab**^	**0.56 ± 0.03** ^**a**^	**0.63 ± 0.03** ^**b**^	0.39 ± 0.02^**a**^	0.39 ± 0.02^**a**^	0.41 ± 0.02^**a**^	0.48 ± 0.04^**a**^	0.48 ± 0.03^**a**^	0.48 ± 0.04^**a**^	0.62 ± 0.03^**a**^	0.57 ± 0.03^**a**^	0.62 ± 0.03^**a**^
	IV	**0.18 ± 0.02** ^**a**^	**0.20 ± 0.02** ^**ab**^	**0.21 ± 0.02** ^**b**^	**1.01 ± 0.03** ^**ab**^	**1.10 ± 0.05** ^**a**^	**1.01 ± 0.05** ^**b**^	0.41 ± 0.03^**a**^	0.46 ± 0.03^**a**^	0.47 ± 0.03^**a**^	**0.35 ± 0.03** ^**a**^	**0.43 ± 0.03** ^**b**^	**0.37 ± 0.03** ^**a**^
	RA	**0.33 ± 0.03** ^**a**^	**0.26 ± 0.03** ^**b**^	**0.32 ± 0.03** ^**ab**^	0.62 ± 0.03^**a**^	0.57 ± 0.03^**a**^	0.63 ± 0.03^**a**^	0.44 ± 0.03^**a**^	0.40 ± 0.03^**a**^	0.42 ± 0.03^**a**^	0.51 ± 0.03^**a**^	0.46 ± 0.03^**a**^	0.51 ± 0.03^**a**^
	CFI	**0.61 ± 0.02** ^**ab**^	**0.57 ± 0.02** ^**a**^	**0.62 ± 0.02** ^**b**^	0.50 ± 0.03^**a**^	0.47 ± 0.03^**a**^	0.51 ± 0.03^**a**^	0.57 ± 0.03^**a**^	0.55 ± 0.02^**a**^	0.56 ± 0.02^**a**^	0.65 ± 0.03^a^	0.60 ± 0.02^a^	0.65 ± 0.02^a^
	I < O	**---**	**---**	**---**	83.88 ± 3.22^a^	78.26 ± 2.93^a^	80.35 ± 2.63^a^	**---**	**---**	**---**	91.61 ± 3.10^a^	88.74 ± 2.89^a^	90.71 ± 2.19^a^
MEN (n = 11)	IS	0.56 ± 0.05^a1^	0.53 ± 0.05^a1^	0.63 ± 0.05^a1^	**0.32 ± 0.02** ^**a1**^	**0.36 ± 0.02** ^**ab1**^	**0.40 ± 0.04** ^**b1**^	0.43 ± 0.07^ª1^	0.46 ± 0.04^a1^	0.47 ± 0.06^a1^	0.55 ± 0.04^a1^	0.52 ± 0.04^a1^	0.58 ± 0.06^a1^
	IV	**0.15 ± 0.02** ^**a1**^	**0.18 ± 0.03** ^**ab1**^	**0.22 ± 0.02** ^**b1**^	1.12 ± 0.04^a1^	1.17 ± 0.08^a1^	1.09 ± 0.08^a1^	0.45 ± 0.04^a1^	0.49 ± 0.06^a1^	0.55 ± 0.05^a1^	0.45 ± 0.04^a1^	0.50 ± 0.06^a1^	0.46 ± 0.04^a1^
	RA	0.34 ± 0.04^a1^	0.28 ± 0.03^a1^	0.30 ± 0.03^a1^	0.51 ± 0.05^a1^	0.55 ± 0.05^a1^	0.55 ± 0.06^a1^	0.41 ± 0.06^a1^	0.40 ± 0.05^a1^	0.41 ± 0.05^a1^	0.44 ± 0.05^a1^	0.44 ± 0.05^a1^	0.48 ± 0.05^a1^
	CFI	0.61 ± 0.03^a1^	0.58 ± 0.04^a1^	0.61 ± 0.03^a1^	0.42 ± 0.03^a1^	0.44 ± 0.05^a1^	0.47 ± 0.05^a1^	0.54 ± 0.05^a1^	0.53 ± 0.05^a1^	0.53 ± 0.04^a1^	0.59 ± 0.04^a1^	0.57 ± 0.05^a1^	0.61 ± 0.05^a1^
	I < O	**---**	**---**	**---**	75.04 ± 5.95^a1^	75.00 ± 5.76^a1^	76.26 ± 3.99^a1^	**---**	**---**	**---**	84.51 ± 6.19^a1^	85.61 ± 5.65^a1^	85.98 ± 3.77^a1^
WOMEN (n = 13)	IS	0.59 ± 0.06^ª1^	0.58 ± 0.04^a1^	0.64 ± 0.04^a1^	0.44 ± 0.02^a2^	0.41 ± 0.02^a1^	0.42 ± 0.03^a1^	0.53 ± 0.04^a1^	0.50 ± 0.03^a1^	0.48 ± 0.05^a1^	0.68 ± 0.04^a2^	0.61 ± 0.03^a1^	0.65 ± 0.04^a2^
	IV	0.20 ± 0.03^a1^	0.22 ± 0.03^a1^	0.19 ± 0.03^a1^	0.92 ± 0.03^a2^	1.03 ± 0.05^a1^	0.93 ± 0.05^a1^	0.37 ± 0.03^a1^	0.44 ± 0.03^a1^	0.41 ± 0.03^a2^	**0.27 ± 0.03** ^**a2**^	**0.37 ± 0.03** ^**b2**^	**0.30 ± 0.02** ^**a2**^
	RA	**0.32 ± 0.04** ^**a1**^	**0.24 ± 0.03** ^**b1**^	**0.33 ± 0.03** ^**a1**^	**0.71 ± 0.03** ^**a2**^	**0.60 ± 0.05** ^**b1**^	**0.69 ± 0.03** ^**ab2**^	0.47 ± 0.04^a1^	0.41 ± 0.04^a1^	0.44 ± 0.04^a1^	**0.57 ± 0.03** ^**a2**^	**0.47 ± 0.03** ^**b1**^	**0.54 ± 0.04** ^**ab1**^
	CFI	0.60 ± 0.04^a1^	0.57 ± 0.03^a1^	0.62 ± 0.03^a1^	**0.56 ± 0.03** ^**a2**^	**0.50 ± 0.03** ^**b1**^	**0.55 ± 0.03** ^**ab1**^	0.60 ± 0.04^a1^	0.57 ± 0.03^a1^	0.57 ± 0.04^a1^	**0.70 ± 0.03** ^**a2**^	**0.63 ± 0.03** ^**b1**^	**0.68 ± 0.03** ^**ab1**^
	I < O	**---**	**---**	**---**	**91.36 ± 1.25** ^**a2**^	**81.02 ± 2.39** ^**b1**^	**83.82 ± 3.34** ^**ab1**^	**---**	**---**	**---**	**97.62 ± 0.65** ^**a2**^	**91.39 ± 2.41** ^**b1**^	**94.72 ± 2.02** ^**ab2**^

Repeated-measures ANOVA and *post hoc* Bonferroni were performed to verify the differences among periods for the whole group or for each sex. Statistical significance was set at *p* < 0.05

Different letters indicate statistically significant differences between periods (also highlighted in bold.) Different numbers indicate significant differences between men and women for each variable and studied period

**Fig. 3:**
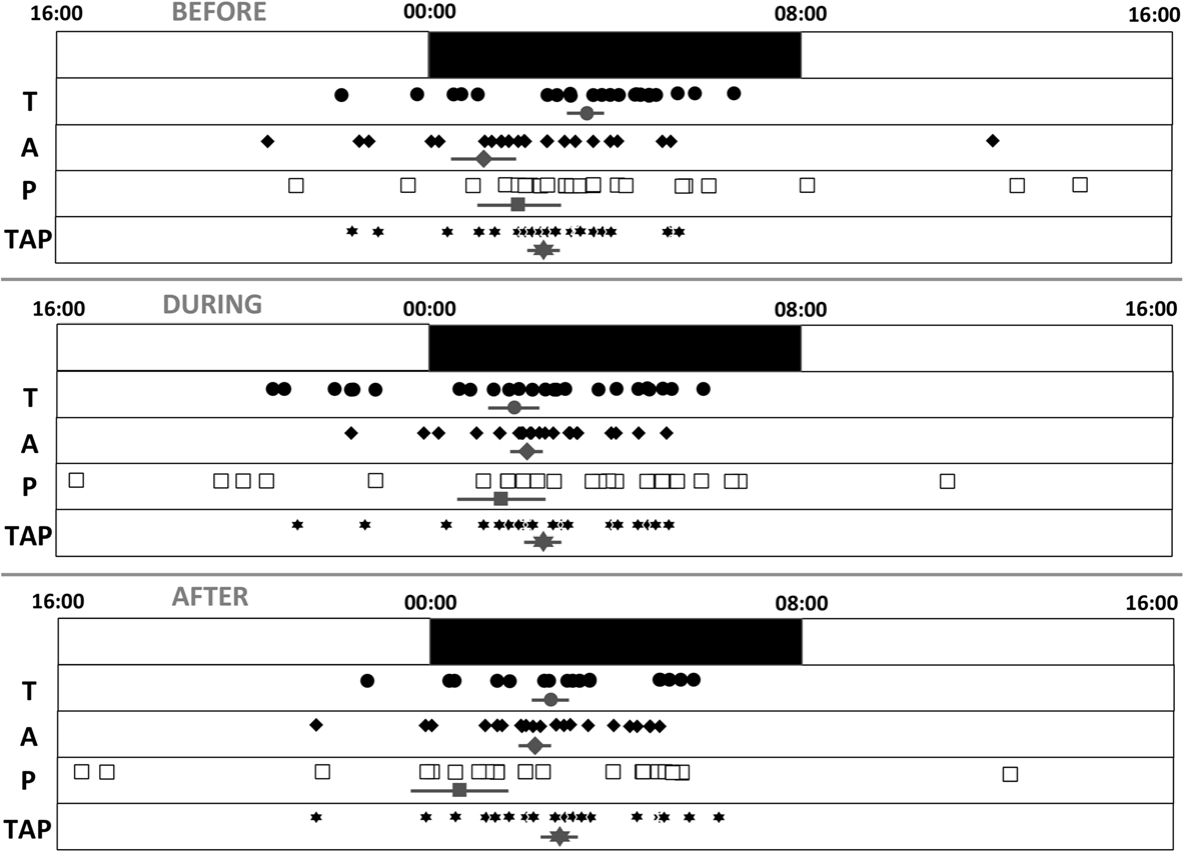
Distribution of phase markers. Individual values (*n* = 24) for M5 (central timing for the 5 consecutive hours of maximal values), in the case of temperature (*T*), or L5 (central timing for the 5 consecutive hours of minimal values) for the remaining variables (activity (*A*), position (*P*) and *TAP*) in each period studied (before, during and after chemotherapy) are represented as black circles, black rhombus, white squares and black stars, respectively. The standard nighttime period is drawn as a black bar between midnight and eight a.m.. In addition to individual values, the mean ± SEM for each variable is also drawn in grey

Individual phase markers ranged from 22:10 to 06:30 for temperature, from 22:30 to 12:10 for activity, from 21:10 to 14:00 for body position and from 22:20 to 05:20 for TAP, thus revealing a difference of up to 12 h among patients with extreme timings (Fig. 3).

Differences could be observed between women and men as early as at baseline, with women always showing stronger and less fragmented rhythms (Table 2). Thus, IS (*p* = 0.002), IV (*p* = 0.001), RA (*p* = 0.001), CFI (*p* < 0.001) and I < O (p = 0.008) for activity, and IV (*p* = 0.002), RA (*p* = 0.032), CFI (*p* = 0.021) and I < O (*p* = 0.032) for TAP were significantly different between women and men.

### Circadian biomarkers during chronotherapy and post-treatment

Overall, the fixed chronotherapy protocols, together with non-time specified monoclonal antibodies for half of the patients, transiently altered the baseline 24-h patterns in all three circadian biomarkers and the integrated TAP (Fig. 1). Treatment-induced changes included a statistically significant increase in rhythm fragmentation (high IV) and lower robustness (low CFI), as compared to baseline values, for temperature, rest-activity, position and TAP (Table 2 and Fig. 2). Similar trends were found for rhythm stability (IS) and amplitude (RA), although the differences were not statistically significant. Complete recovery was evidenced by data inspection after the course of chronotherapy.

### Evaluation of circadian robustness

Circadian robustness was evaluated by using the CFI from the composite variable TAP, which was previously validated in [26]. Furthermore, we studied the coherence of phase markers for wrist temperature and activity rhythms throughout the study using a new index, DI or Desynchronisation Index, which quantifies timing differences between two phase markers, M5 for wrist temperature and L5 for activity, which should be in phase in healthy subjects with proper internal temporal order.

**Fig. 4:**
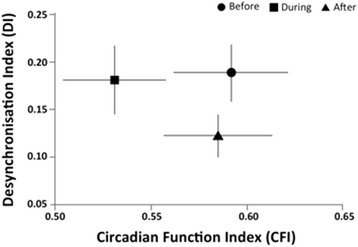
Relationship between the degree of desynchronization and circadian rhythm robustness (*n* = 24). Relationship between the internal temporal order desynchronization index (DI for temperature and activity) and robustness of circadian rhythms (CFI for the composite variable TAP) before (circle), during (square) and after (triangle) chronotherapy in cancer patients. Data are expressed as mean ± SEM

**Fig. 5:**
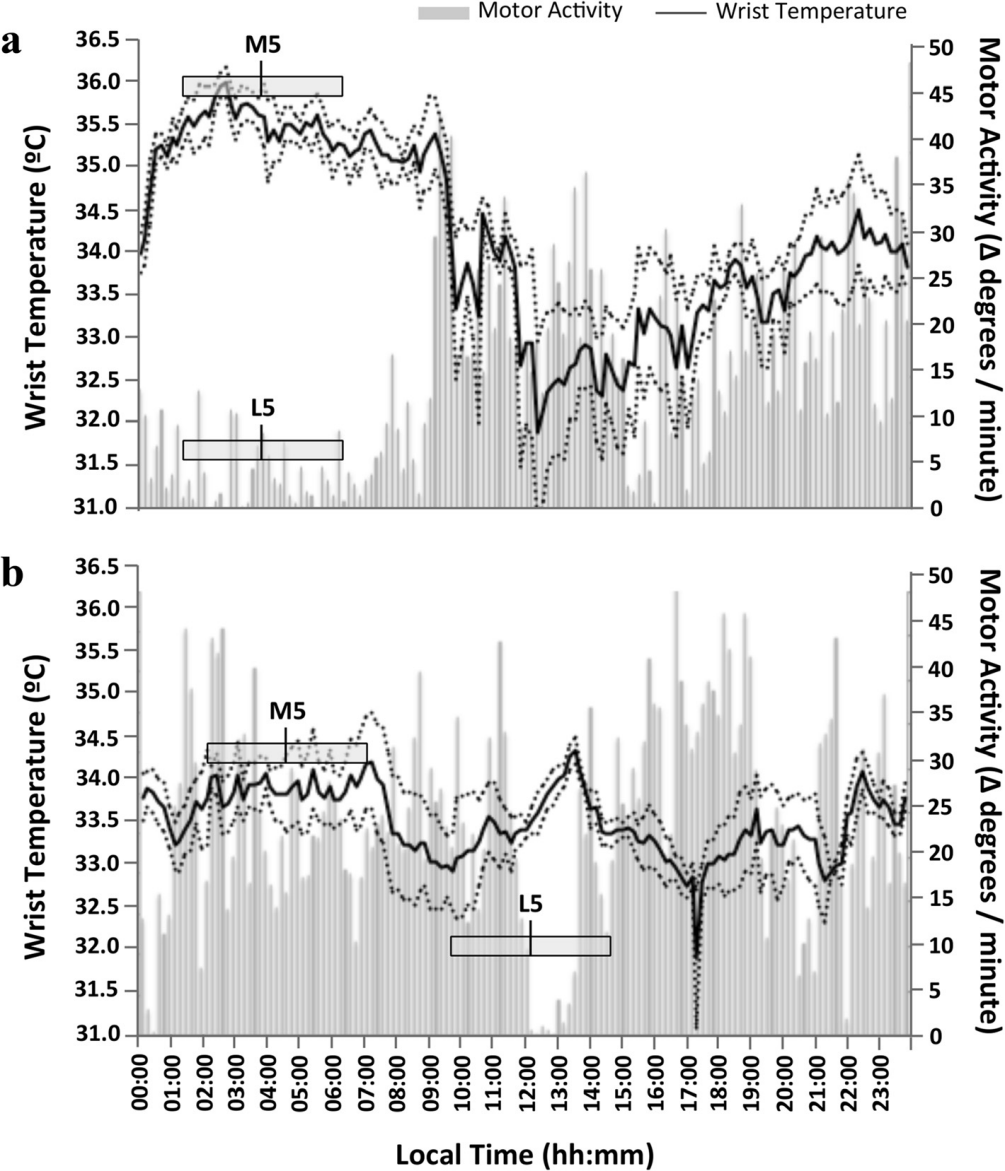
Wrist temperature and activity pattern for two patients, one with a low DI between wrist temperature and motor activity rhythms (**a**) and other with a high DI (**b**). Mean waveforms for the baseline pre-chemo period (4 days) are represented. Wrist temperature rhythm is drawn as a solid black line and motor activity is shown as grey bars. In addition, the five consecutive hours of maximal and minimal values for temperature and motor activity and their central timing (M5 and L5, respectively) are indicated by grey rectangles. Wrist temperature error (the standard error of the mean, SEM) is represented by dotted lines above and below the wrist temperature series. Motor activity SEM is omitted in this figure for reasons of clarity. **a** corresponds to a patient with a DI = 0.00; note that M5 = 03:50 h and L5 = 03:50 h coincide. **b** corresponds to a patient with a DI = 0.63; note not only the difference in timing for M5 = 04:40 h; L5 = 12:10 h, but also the lack of any overlapping

Figure 4 shows that the administration of chemotherapy decreased the robustness of the circadian system (lower CFI values during *versus* before chemotherapy), as explained by the perturbation of the wrist temperature, motor activity and body position rhythms, while a similar degree of desynchronization between temperature and motor activity was maintained (similar DI values before and during chemotherapy). However, the shock of chemotherapy drugs administered in a chronomodulated fashion seemed to produce the re-synchronization of at least temperature and activity rhythms, and a reinforcement of circadian robustness, which was achieved after the administration of chemotherapy (Fig. 4). An example of this disruption of the temporal internal order can be found in Fig. 5, which shows temperature and activity rhythms for two patients.

### Sex-dependent response of circadian biomarkers to the fixed chronotherapy protocol

The fixed chronotherapy protocols resulted in a significantly greater deterioration of TAP rhythms in women than in men, despite the fact that baseline rhythms were found to be more robust in women (Table 2). However, this sex-dependent effect was not consistent for every studied variable, probably due to the small sample size.

### Toxicity

A single patient displayed grade 4 neutropenia. No other hematological or clinical grade 3 or 4 toxicity was encountered. The most frequent adverse events consisted of grade 1 or 2 diarrhea (67 % of the patients), fatigue (58 %), anorexia (46 %), mucositis (46 %) and peripheral sensory neuropathy (46 %).

A body weight loss >5 % and a fatigue ≥ grade 2 were experienced by 5 and 9 patients, respectively, following chronotherapy. Baseline CFI and DI did not predict the occurrence of any toxicity. Interestingly, those patients with this clinically relevant weight loss or fatigue displayed a higher DI during chronotherapy (median = 0.22), as compared to the patients without such toxic symptoms (median = 0.10; *p* = 0.04).

## Discussion

This study has shown for the first time that in cancer patients the circadian system presents a huge variability in terms of phase distribution (12 hour spread among different patients) (Fig. 3) and more importantly, a moderately perturbed temporal internal order (Fig. 4). This was evaluated through the concomitant and continuous measurement of 3 different circadian biomarkers (T, A, P). This monitoring is feasible in cancer patients receiving chemotherapy on an outpatient basis, using non-invasive devices that are readily tolerated by patients for 12 days, without significant complaints. Moreover, we observed that the chronomodulated administration of chemotherapy affected the robustness of the circadian system, increasing fragmentation and diminishing the stability of wrist temperature, motor activity and body position rhythms during the treatment (Table 2). Surprisingly, the chronochemotherapy was able to partially resynchronize the rhythms studied, leading to reduced perturbation of the internal temporal order after the treatment.

In cancer patients, the rest-activity rhythm has been reported to be altered even before the administration of chemotherapy [4, 5, 37, 38]. Previous studies have shown that chemotherapy induces rapid-onset and sustained alterations in the rest-activity rhythm in most cancer patients [12, 37, 39, 40]. The present study confirms this finding and extends these results to wrist temperature, body position and TAP rhythms, which were also perturbed to a large extent by chemotherapy.

The consequences of a disturbed circadian system in cancer patients have been previously described with respect to rest-activity and cortisol rhythms. In this sense, having a robust rest-activity rhythm increases overall survival [3–5]. Furthermore, a flattened cortisol rhythm or disturbed sleep pattern in cancer patients is linked to a higher risk of early death and shorter overall survival [41–47]. Given the importance of these facts, future studies should specifically evaluate the effect of diminishing disruption, including sleep disruption, on survival in cancer.

A challenged internal temporal order, as observed after long-haul transmeridian flights or rotating or night shift work, increases the risk of developing certain types of cancer, diabetes or metabolic syndrome and cardiovascular diseases [48, 49]. However, no data to date are available on the internal temporal order of cancer patients. Here, to the best of our knowledge, we provide the first evaluation of the internal synchronization of patients already suffering from advanced cancer. In this study, we were able to quantify the internal rhythmic coherence of our patients throughout a course of chemotherapy. We found that, in the presence of moderate desynchronization at baseline, chronomodulated chemotherapy was, on average, able to partially re-synchronize these rhythms by the end of the study. This demonstrates the usefulness of circadian-based chemotherapy, at least in reducing desynchrony among the internal rhythms in these patients. However, the limited number of patients enrolled in this study prevent for definitive conclusions on the clinical outcomes of prolonged internal desynchronization. Thus, more extensive future studies should address the implications of this finding on the survival rates of these patients.

Sex differences in the effect of chronomodulated treatments for colorectal cancer have previously been described [13, 14]. Our results showed some rhythmic differences according to sex but not enough to draw any definitive conclusions. Comprehensive studies with a larger sample of patients would be needed to that effect.

The large inter-individual differences, up to 12 hours, among patients already observed at baseline were also found consistently throughout the study, both during and immediately after the administration of chemotherapy. This variability could potentially affect the safety and efficacy of cancer treatments. The administration of chemotherapy schedules in a chronomodulated fashion attempts to find the optimal timing of maximum antitumor activity and minimum side effects for the host [11], which has been proven effective in both murine models (mostly male mice) [11, 50] and humans [3, 7, 51]. Up to now, these schedules have been administered without considering the individual phase of each patient’s rhythms, and thus the hypothesis of administration at the optimal moment is challenged. Therefore, this study establishes the proof of principle for future studies focused on the individualization of cancer treatment, based not only on population studies, but also on individual circadian rhythm phases.

Evidence exists in the literature regarding the benefits of chronoenhancement in cancer patients, either through the administration of melatonin [52], or by optimally-timed exposure to bright light [53]. Therefore, other circadian therapeutic approaches, including sleep hygiene and regular feeding schedules, could be implemented in order to restore circadian function or to prevent chemotherapy-induced circadian disruption. In mice, for example, circadian amplification of the core body temperature rhythm through meal timing has been observed to cut experimental cancer growth in half [54]. These chronoenhancement techniques could potentially be useful to strengthen and synchronize cancer patients’ circadian system prior to chemotherapy administration, especially for those patients with persistent circadian disruption. This methodology would hypothetically, on the one hand, find the optimal time for their treatment and on the other hand, maximize treatment efficacy once administered at the right individual time.

## Conclusions

In summary, both experimental and clinical data support the relevance of a robust circadian system in order to enhance the efficacy and tolerability of chronomodulated treatments. Thus, reliable, non-invasive and continuously assessed circadian biomarkers, such as those provided by the rest-activity, body position and temperature monitoring computed in the integrated variable TAP are required to optimize cancer treatments, taking into account the status and phase of individual circadian systems. The great inter-patient variability at baseline, during and after treatment, and the differing profound effects of chemotherapy on circadian robustness, phase and internal order synchronization confirm the interest of such multiparametric evaluation of cancer outpatients. The useful information provided by this concomitant TAP monitoring is also relevant for interventional studies targeting the circadian timing system, in order to enhance or protect its function, with the aim of improving the wellbeing and outcomes of cancer patients.

## Abbreviations

TAP: integrated variable composed of wrist Temperature, motor Activity and body Position
I < O: dichotomy index In bed < Out of bed
DI: Desyncronization Index
SCN: SupraChiasmatic Nuclei of the hippothalamus
CBT: Core Body Temperature
DLMO: Dim Light Melatonin Onset
CTS: Circadian Timing System
PS: Performance Status
W.H.O.: World Health Organization
5-FU-LV: 5-Fluorouracil
CPT-11: Irinotecan
NCI CTC-AE v3.0: National Cancer Institute’s Common Terminology Criteria for Advance Events version 3.0
VL5: mean value of five consecutive hours with the lowest values
L5: mean timing of five consecutive hours with the lowest values
VM10: mean value of ten consecutive hours with the highest values
M10: mean timing of ten consecutive hours with the highest values
VM5: mean value of five consecutive hours with the highest values
M5: mean timing of five consecutive hours with the highest values
RA: Relative Amplitude
IS: Interdaily Stability
IV: Intradaily Variability
CFI: Circadian Functioning Index
ANOVA: ANalysis Of VAriance
SEM: Standard Error of the Mean
CT: chemotherapy
y.o.: years old
